# Prevalence of Antimicrobial Use in Small Animal Dermatology Referral Practices in the United States

**DOI:** 10.1111/vde.70087

**Published:** 2026-05-27

**Authors:** Domenico Santoro, Emma R. Bollig, Jennifer L. Granick

**Affiliations:** 1 Department of Small Animal Clinical Sciences, College of Veterinary Medicine, University of Florida, Gainesville, Florida, USA; 2 Department of Veterinary Clinical Sciences, College of Veterinary Medicine, University of Minnesota, St Paul, Minnesota, USA

**Keywords:** antimicrobial stewardship, antimicrobial use, cats, companion animal, dermatology, dogs

## Abstract

**Background:**

Antimicrobial resistance is a critical global health concern driven by antimicrobial use. Dermatological conditions are common reasons for antimicrobial prescriptions in small animal practice, yet data on prescribing patterns in veterinary dermatology referral settings are lacking.

**Hypothesis/Objectives:**

To characterise the frequency, types and indications for antimicrobial use in dogs and cats seen by board-certified or residency-trained first-opinion veterinary surgeons in the United States.

**Animals:**

Medical record data about 550 client-owned animals (479 dogs, 71 cats) presenting to 22 US veterinary dermatology referral practices were included.

**Materials and Methods:**

A prospective, cross-sectional point-prevalence study was conducted in August 2023. Each clinic enrolled the first 25 consecutive dogs or cats evaluated by a referral clinician. Data collected included clinical diagnoses, antimicrobial and antiseptic prescriptions and diagnostic testing.

**Results:**

Antimicrobials were prescribed in 54.9% (302 of 550) of cases. Third-generation cephalosporins accounted for 43.9% (54 of 123) of systemic antibiotics to dogs. Of topical antimicrobials prescribed for otitis externa associated with bacteria only or yeast only, 36.4% (20 of 55) were a combination formulation containing both an antibiotic and an antifungal. Antiseptics were prescribed in 62.7% of cases. Cytological investigation was performed in 86.3% of conditions treated with antimicrobials, whereas bacterial culture was used in only 15.3%.

**Conclusions and Clinical Relevance:**

This study provides a benchmark for antimicrobial use in veterinary dermatology referral practices. Findings highlight opportunities to align prescribing with updated guidelines to reduce reliance on broad-spectrum systemic antibiotics, favouring topical therapies and underscore the need for narrow-spectrum topical options.

## 1 | Introduction

Antimicrobial resistance (AMR) has been recognised by the World Health Organization as one of the top global public health threats [1]. This threat is present in companion animal veterinary practice, where the incidence of AMR infections is increasing [2]. Isolates of *Staphylococcus pseudintermedius*, the main opportunistic pathogen causing pyoderma in dogs, are rarely pan-susceptible and often multidrug-resistant, making treatment challenging [3]. Efforts to combat AMR include optimisation of antimicrobial use and reduction of inappropriate use, as all antimicrobial use is a controllable risk factor for the selection of antimicrobial-resistant organisms.

Understanding what and how antimicrobials are used is key to modifying antimicrobial use practices. Antimicrobial stewardship (AS) has been defined by the American Veterinary Medical Association (AVMA) as ‘the actions that veterinarians take, both as an individual and as a profession, to preserve the effectiveness and availability of antimicrobial drugs through conscientious oversight and responsible decision-making while safeguarding animal, public, and environmental health’ [4]. Evaluation of antimicrobial drug use practices is one of AVMA’s five core principles of AS. Measurement of antimicrobial use allows for establishment of baseline prescribing, identification of goals for improvement and tracking progress over time. Sharing of antimicrobial use data with prescribers highlights the veterinary surgeon’s role in preserving the effectiveness of these drugs through oversight and judicious decision-making.

It has been hypothesised that the probability of prescribing systemic antimicrobials differs between primary care, emergency and critical care, and specialty referral services. In a series of point-prevalence studies of antibiotic use in various practice settings, the prevalence of antibiotic prescriptions for dogs and cats among US veterinary teaching hospitals and non-academic referral hospitals was in the ranges 31.4%–36.5% for dogs and 26.4%–27.3% for cats in primary care settings [5, 6]. In another study evaluating antimicrobial use in three US veterinary teaching hospitals, dogs and cats presenting to emergency or speciality services were more likely to be prescribed antimicrobials compared to primary care patients [7].

Skin and ear diseases are among the most common conditions for which small animals are presented to a veterinary surgeon [8, 9]. These conditions also are among the ones for which antimicrobials are most often prescribed to dogs and cats [5, 7, 10–12]. No study has been published addressing the frequency of antibiotic prescribing in dermatology speciality practices, including the utilisation of topical versus systemic treatment. Therefore, the aim of this study was to describe antimicrobial use in veterinary referral dermatology practices. The primary objectives were to measure the frequency of antimicrobial prescribing for dogs and cats presenting to US veterinary referral dermatology practices and the most common antimicrobial drugs prescribed, indications for use, adjunctive therapies and associated evidence of bacterial infection.

## 2 | Materials and Methods

### 2.1 | Clinic Enrolment and Ethics Statement

Small animal clinics were recruited via the American College of Veterinary Dermatology (ACVD), ACVD specialty board communication, a small animal AS listserv, direct emails to previous antibiotic-use study participants [5, 6], and emails to state veterinary medical associations from June to August 2023. Clinics interested in participating were evaluated so that only those in the United States with residency-trained or board-certified first-opinion veterinary surgeons seeing dogs and cats were included. Participating practices obtained any approval necessary by clinic leadership.

With a scope limited to collection of veterinary medical data, the study was determined exempt from review by the University of Minnesota Institutional Animal Care and Use Committee (IACUC). This study was categorised as ‘not human research’ by the University of Minnesota Institutional Review Board (IRB).

### 2.2 | Data Collection, Management and Analysis

A facility coordinator(s) (e.g., veterinary surgeon, veterinary technician) was identified at each participating clinic and completed a survey about their clinic’s AS and infection prevention and control practices and clinical approach to select dermatological infections in consultation with the veterinary team. The survey is available in Appendix S1 in the Supporting Information. It was provided to the facility coordinator on 19 July 2023 and was completed before data entry.

Beginning 1 August 2023, the first 25 canine and/or feline patients presenting to a residency-trained or board-certified first-opinion veterinary surgeon were included, regardless of whether the visit resulted in an antimicrobial prescription. Data collected included signalment; a maximum of three dermatological clinical conditions (e.g., cutaneous, otic) and, if available, a specific diagnosis (e.g., atopic dermatitis (AD), deep pyoderma); diagnostic testing performed (Appendix S2); the name of antiseptics prescribed; and the name, route and duration of antimicrobials (antibiotics, antifungals and antibiotic–antifungal combination drugs) prescribed. Diagnostic results available before the appointment date and used for clinical decision-making on the study date were included.

Antimicrobials were considered unique based on the established (non-proprietary) drug name for patient-level analyses. If a patient was prescribed two formulations of the same antimicrobial (e.g., intravenous clindamycin transitioned to oral clindamycin), these were considered a single antimicrobial drug. Alternatively, if a patient was prescribed two chemically distinct antimicrobials (e.g., intravenous ampicillin-sulbactam transitioned to oral amoxicillin-clavulanic acid), these were considered two antimicrobial drugs. If a referring clinician prescribed the patient an antimicrobial and the consulting clinician made the decision to continue the antimicrobial, that drug was included; if a prescription made by the referring clinician was not continued, it was not included. If a patient was prescribed multiple antimicrobials, details of each were recorded. Antimicrobials intended to treat multiple clinical conditions in a single patient were associated with each condition. For each antimicrobial, the intended reason for use (treatment of infection, prophylaxis, non-antimicrobial effects and undetermined) was indicated. Criteria for determining the level of evidence of infection are available in Table S1. The same antimicrobial could be prescribed to a patient for more than one clinical condition with different intended reasons for use. Antiseptics were considered unique based on the active ingredient list. Antiseptics that included an antifungal drug were analysed as antiseptics and not included in counts of antimicrobial drugs.

All data were entered into a secure online data collection tool (REDCap) [13] by 29 October 2023. Analyses were performed using SAS (release 9.4, 1997; SAS Institute). The data were summarised as frequencies and percentages. Chi-squared tests and logistic regression were used to evaluate univariate and multivariate associations, respectively. Statistical significance was determined at *p* < 0.05.

## 3 | Results

### 3.1 | Participating Practices

Twenty-two dermatology practices participated in the study, including eight (36.4%) veterinary teaching hospitals and 14 (63.6%) non-academic referral clinics. All geographical regions [14] were represented: eight clinics in the South, five in the Midwest, five in the Northeast and four in the West. A majority of clinics (72.7%, 16 of 22) indicated that they utilise published small animal antibiotic-use guidelines for specific conditions, such as the International Society for Companion Animal Infectious Diseases guideline for superficial bacterial folliculitis in dogs [15]. Half of clinics (11 of 22) reported that they take a formal approach to AS, with over one-third (36.4%, eight of 22) having an established programme, protocol or policy, and seven (31.8%) having a formal AS committee. Major barriers to taking action regarding AS included lack of staff time dedicated to AS activities (50.0%, 11 of 22), lack of formal commitment or interest from clinic staff (22.7%, five of 22) and uncertainty of how to establish an AS committee (22.7%, five of 22). Six (27.3%) clinics indicated that they have no barrier to taking action regarding AS. Key factors identified by respondents as important for optimisation of antimicrobial use in veterinary dermatology were alternatives to antimicrobials for common conditions (90.9%, 20 of 22), duration of antimicrobial use (86.4%, 19 of 22), client communication support (63.6%, 14 of 22), and specific antibiotic selection recommendations for common conditions (59.1%, 13 of 22). Fourteen (63.6%) clinics take a formal approach to infection prevention and control, with an established programme, protocol or policy.

Condition-specific questions also were posed to referral clinicians in the pre-study survey. While the majority (72.7%, 16 of 22) of the clinicians indicated that they would avoid systemic antimicrobials and start with topical therapies for cases of surface pyoderma, a minority (22.7%, five of 22) would take the same approach for superficial pyoderma; preferred antibiotic selections for superficial pyoderma were cephalexin (27.3%, 6 of 22), clindamycin (27.3%, 6 of 22) and third-generation cephalosporins (18.2%, 4 of 22). Most indicated that they would perform culture and susceptibility testing for cases of deep pyoderma caused by coccoid-shaped bacteria (72.7%, 16 of 22) or rod-shaped bacteria (95.4%, 21 of 22).

### 3.2 | Study Population

A total of 550 animals were included in the study, including 200 from veterinary teaching hospitals and 350 from non-academic referral clinics. Most were dogs (87.1%, 479 of 550) and 12.9% (71 of 550) were cats. There was no difference between species (*p* = 0.27), neuter status (*p* = 0.54) or median age (*p* = 0.18) between patients evaluated at veterinary teaching hospitals and non-academic referral clinics. Additional demographic characteristics for dogs and cats, including sex, age, comorbidities, immunocompromising drugs and weight, are presented in Table S2.

### 3.3 | Antimicrobial Drugs Prescribed

At least one antimicrobial was prescribed in 50.0% (100 of 200) of patients enrolled through veterinary teaching hospitals and in 57.7% (202 of 350) patients enrolled through non-academic referral clinics; there was no significant difference in the frequency of antimicrobial prescribing between these two settings (chi-square: *p* = 0.08). Antimicrobial prescribing frequency for dogs was 58.7% (281 of 479) and for cats was 29.6% (21 of 71), with an overall frequency of 54.9% (302 of 550). This represented a total of 443 antimicrobial drugs, including 244 antibiotics, 122 antifungals and 77 antibiotic–antifungal combination prescription drugs. Of all antimicrobial drugs, 46.7% (207 of 443) were prescribed for systemic administration, and 53.3% (236 of 443) were prescribed for topical application. Patients were prescribed, administered or continued one (61.6%, 186 of 302), two (30.8%, 93 of 302), three (7.0%, 21 of 302) and four (0.7%, two of 302) antimicrobial drugs.

Antibiotics were prescribed, administered or continued for 37.3% (205 of 550) of patients, 39.7% (190 of 479) of dogs and 21.1% (15 of 71) of cats. Among the 244 antibiotic drug prescriptions, 54.9% (134 of 244) were for systemic administration and 45.1% (110 of 244) were for topical application. The most common classes of antibiotics for systemic administration for dogs were third-generation cephalosporins (43.9%, 54 of 123), with cefpodoxime proxetil (39.0%, 48 of 123) for oral administration being the most selected in that class, and lincosamides (19.5%, 24 of 123), with clindamycin (19.5%, 24 of 123) for oral administration being the only antibiotic selected in that class (Table 1). Dogs were prescribed cefpodoxime proxetil or clindamycin for a median of 30 days with a range of 7–45 days and 5–60 days, respectively. The most common class of antibiotics for systemic administration in cats was third-generation cephalosporins (63.6%, 7 of 11), including five prescriptions for cefovecin and two for cefpodoxime proxetil. In cats, the median duration for cefpodoxime was 22 days (range 14–30) and 14 days (range 14–30) for cefovecin.

Antifungals were prescribed, administered or continued for 20.4% (112 of 550) of patients, 22.3% (107 of 479) of dogs and 7.0% (five of 71) of cats. Among the 122 antifungal drug prescriptions, 59.8% (73 of 122) were for systemic administration, and 40.2% (49 of 122) were for topical application. The most common classes of antifungals for systemic administration for dogs were imidazoles (55.6%, 40 of 72), with ketoconazole (55.6%, 40 of 72) being the only antifungal selected in that class, and first-generation triazoles (34.7%, 25 of 72), with fluconazole (30.6%, 22 of 72) being the most selected in that class (Table 2). Dogs were prescribed ketoconazole and fluconazole for a median of 30 days with a range of 14–383 days and 14–582 days, respectively. There was a single systemic antifungal prescription, fluconazole, for cats.

Antibiotic–antifungal combination drugs were prescribed, administered or continued for 12.9% (71 of 550) of patients, 14.0% (67 of 479) of dogs and 5.6% (4 of 71) of cats. All antibiotic–antifungal combination drugs were for topical application (100%, 77 of 77) (Table 3).

The majority (91.9%, 407 of 443) of antimicrobial prescriptions were intended for the treatment of infection. Of prescriptions for infection treatment, 92.4% (377 of 408) were for patients with confirmed infections, 6.9% (28 of 408) were suspected infections, and 0.7% (3 of 408) had no evidence of infection. The most frequent antimicrobials prescribed for the treatment of infection were cefpodoxime proxetil (12.0%, 49 of 407), enrofloxacin (8.6%, 35 of 407) and ketoconazole (8.1%, 33 of 407). A minority of antimicrobial prescriptions were intended for reasons other than the treatment of infection, including 4.3% (19 of 443) for non-antimicrobial effects. Of these, 47.4% (9 of 19) were ketoconazole prescriptions, and 26.3% (5 of 19) were prescriptions of doxycycline, both for dogs. Of the nine prescriptions of ketoconazole for non-antimicrobial effects, all were prescribed for skin conditions (one pemphigus foliaceus, four dermatitis-atopic, one deep pyoderma, three dermatitis-allergy of unknown aetiology). The five prescriptions of doxycycline for non-antimicrobial effects were used to treat six conditions; three were for skin conditions (two other, one open diagnosis), one for mucocutaneous pyoderma, one for discoid lupus erythematosus of the nasal planum and one for perianal fistula. No cats received antimicrobials for non-antimicrobial effects. For prophylactic purposes (3.8%, 17 of 443), 11 different antimicrobials were prescribed, the most common of which was miconazole (29.4%, 5 of 17). All prescriptions had an identified reason for use.

### 3.4 | Use of Antiseptic Drugs

Antiseptic drugs were prescribed for 68.3% (327 of 479) of dogs and 25.4% (18 of 71) of cats, for a total frequency of 62.7% (345 of 550). Nearly one-in-four (24.9%, 137 of 550) animals were prescribed at least one antiseptic with no accompanying antimicrobial prescription, 17.1% (94 of 550) were prescribed at least one antimicrobial without an antiseptic prescription, 37.8% (208 of 550) were prescribed at least one antimicrobial and at least one antiseptic, and 20.2% (111 of 550) were not prescribed either an antimicrobial or antiseptic. Of the 345 animals that were prescribed an antiseptic, 61.7% (213 of 345) were prescribed a single antiseptic, 31.6% (109 of 345) were prescribed two antiseptics, 6.4% (22 of 345) were prescribed three antiseptics, and 0.3% (one of 345) was prescribed four antiseptics. There were 501 instances of antiseptic prescriptions for 345 dogs and cats (Table 4). Chlorhexidine-containing antiseptics were the most common antiseptic prescription (58.9%, 295 of 501), with 30.3% (152 of 501) of prescriptions being topical formulations containing chlorhexidine alone. An antifungal was contained in 34.5% (173 of 501) of antiseptic prescriptions. There were no chlorhexidine formulations containing an antibiotic.

### 3.5 | Clinical Conditions

There were 845 clinical conditions addressed for the 550 patients. The most common clinical conditions were classified as cutaneous (56.0%, 473 of 845), otic (28.6%, 242 of 845) and interdigital (9.3%, 79 of 845) (Table S3), with otitis externa (OE) and AD the most common specific diagnoses. Allergy-associated disease represented the most common clinical disease for which animals were seen (49.9%, 422 of 845). Nearly half (45.0%, 380 of 845) of all conditions resulted in at least one antimicrobial prescription (Tables 5 and S4). Over one-third (38.1%, 180 of 473) of cutaneous skin conditions, 61.2% (148 of 242) of otic conditions and 40.5% (32 of 79) of interdigital conditions were prescribed at least one antimicrobial. OE and pyoderma in dogs resulted in the most antimicrobial prescriptions.

### 3.6 | Otitis

Among the 189 animals (174 dogs, 15 cats) with OE, 61.4% (116 of 189) were prescribed a topical antimicrobial, 2.6% (five of 189) were prescribed a systemic antimicrobial, and 67.2% (127 of 189) were prescribed an antiseptic. Among the 35 cases of OE caused by bacteria only (i.e., no yeast identified) and prescribed topical antimicrobials, 34.3% (12 of 35) were treated with a medication containing an antifungal. Among the 20 cases of OE caused by yeast only and prescribed topical antimicrobials, 40.0% (eight of 20) were treated with a medication containing an antibacterial. Among prescriptions for antibiotic-containing topical formulations for dogs with OE, 40.2% (41 of 102) included a fluoroquinolone, 29.4% (30 of 102) contained an aminoglycoside, and 25.7% (16 of 102) contained an amphenicol.

### 3.7 | Pyoderma

There were 100 patients (98 dogs, 2 cats) with pyoderma (deep, superficial focal, superficial generalised, surface, cutaneous dysbiosis/bacterial or yeast overgrowth, cellulitis), excluding interdigital pyoderma. Among the 98 dogs, 19.4% (19 of 98) were prescribed a topical antimicrobial, 45.9% (45 of 98) were prescribed a systemic antimicrobial, and 84.7% (83 of 98) were prescribed an antiseptic (Table 6). Over one-third of dogs with pyoderma (36.7%, 36 of 98) were prescribed an antiseptic solely, and 13.3% (13 of 98) were prescribed a topical antimicrobial in the absence of a systemic antimicrobial. Of the 50 prescriptions for dogs with pyoderma, the most common systemic antibiotics were cefpodoxime proxetil (26.0%, 13 of 50) and clindamycin (22.0%, 11 of 50), whereas only 8.0% (four of 50) were prescribed cephalexin. When systemic antibiotics were prescribed, the duration differed with the type of pyoderma: animals with surface pyoderma were prescribed antimicrobials for a median of 20 days (*n* = 3, range 16–20 days), those with superficial generalised pyoderma for a median of 14 days (*n* = 23, range 14–393 days), those with superficial focal pyoderma for a median of 30 days (*n* = 4, range 24–35 days), and those with deep pyoderma for a median of 31 days (*n* = 15, range 16–60 days).

### 3.8 | Use of Diagnostic Testing to Guide Antimicrobial Prescribing

At least one diagnostic test was performed for 73.1% (402 of 550) of all patients, including 90.3% (363 of 479) of dogs and 54.9% (39 of 71) of cats. Most patients (89.4%, 270 of 302) were prescribed at least one antimicrobial, and 53.2% (132 of 248) of patients not prescribed an antimicrobial had diagnostic testing performed (Appendix S2). Of the 380 clinical conditions identified that resulted in an antimicrobial prescription, 86.3% (328 of 380) had cytological evaluation performed, and 15.3% (58 of 380) had bacterial culture performed.

Among dogs, 68.8% (11 of 16) with deep pyoderma had a bacterial culture and susceptibility performed; corresponding cytological evaluation was done for 10 of 11 of these, and results were available at the time of the consult for eight of these dogs. Cytological results showed that cocci were present in all eight cases, and rods were also present in seven of eight. Culture and susceptibility testing were submitted for 30.4% (14 of 46) of dogs with superficial generalised pyoderma, and none were ordered for the seven dogs diagnosed with surface pyoderma. Of dogs with bacterial OE, 4.4% (7 of 159) had a bacterial culture performed, as did 14.3% (2 of 14) of dogs with otitis media.

Third-generation cephalosporins were prescribed to 65 patients with 72 clinical conditions; 12.3% (8 of 65) of those patients had a bacterial culture and susceptibility performed for the clinical condition associated with the prescription. Fluoroquinolones were prescribed to 50 patients, for a total of 52 clinical conditions; 18.0% (9 of 50) had a culture performed for the clinical condition associated with the prescription.

## 4 | Discussion

The results of this study describe the antimicrobial prescribing patterns in referral dermatology practices in the United States, with over half of patients prescribed antimicrobials to treat bacterial or fungal infections. Referral dermatology practices saw a higher proportion of dogs (87%) than cats (13%), even more skewed than other studies not focussed solely on dermatological conditions seen by speciality or primary care facilities [5, 10, 11]. For both dogs and cats, the most common clinical conditions addressed at dermatology appointments were OE and AD. OE and pyoderma in dogs resulted in the highest number of antimicrobial prescriptions.

The updated ISCAID guidelines emphasise and encourage the use of topical antiseptics as the sole antimicrobial treatment modality for superficial pyoderma, reserving systemic antimicrobials for deep pyoderma or cases of superficial pyoderma unresponsive to topical therapy [16]. This concept strongly reinforced an idea introduced by the previous guidelines available at the time of this study [15]. In this study, topical antimicrobials were infrequently (36.7%) prescribed as a sole therapy for pyoderma and rarely (13.3%) prescribed in the absence of a systemic antimicrobial. Understanding whether barriers to embracing topical treatment alone for pyoderma are clinician- or client-driven is needed to effect practice change.

Topical antimicrobial therapy for OE was frequent in this study. However, a third of OE caused by bacteria only and 40% of those caused by yeast only were treated with topical therapies not targeted solely to the organism type involved. This practice is likely to be dictated by the limited availability of commercial products that are narrow in. spectrum. The use of antiseptic compounds should be considered an alternative to antibiotic–antifungal combinations.

Third-generation cephalosporins were the most prescribed systemic antibiotics. Although most clinics indicated that their preferred systemic antibiotics for superficial pyoderma were cephalexin and clindamycin, data collected about actual prescribing showed that cefpodoxime proxetil was favoured, with clindamycin being the second most prescribed. Although many animals presenting to referral dermatology services have a history of antibiotic use, there is likely to be no benefit of escalating therapy from the first-generation to the third-generation cephalosporin [17]. This correlation is unsurprising given that the *mecA* gene conferring methicillin resistance results in *Staphylococcus* resistance to all beta-lactam antibiotics. Thus, the third-generation cephalosporin is unlikely to be efficacious when resistance to cephalexin is present, and its use should be supported by bacterial culture and susceptibility testing.

In the present study, antibiotics and antifungals were both prescribed for an overall median time of 30 days. Although guidelines for the treatment of superficial pyoderma available at the time of this study do support the use of antibiotics for ≤ 30 days [15], more recent guidelines suggest a reduction in the duration of treatment. Indeed, the newly published guidelines reference evidence that many cases of superficial pyoderma will resolve with 2 weeks of systemic antibiotic therapy or 3 weeks for deep pyoderma; the guidelines strongly recommend re-evaluating patients after 2 weeks to determine whether or not antibiotics should be continued [16]. Unfortunately, there are no guidelines for the duration of antifungal therapy in dermatological patients.

Cytological evaluation was frequently used, yet bacterial culture and susceptibility testing were infrequent, even for deep pyoderma, despite most clinics reporting in the pre-study survey that they would utilise this test for cases of deep pyoderma. Given the accessibility of skin for sampling, utilisation of this test by referral clinicians might be expected to be higher. When culture was used, it was most frequently for deep pyoderma, which could be motivated by higher pathogen diversity and potentially a more unpredictable resistance profile associated with this condition [16]. Expense of testing and cases treated solely with topical antiseptics may have driven low use of culture. Culture and susceptibility panels do not predict efficacy of topical antimicrobials owing to a lack of breakpoints for topical formulations and poor correlation between susceptibility results and clinical response [18–21]. Furthermore, veterinary surgeons have cited cost as a major barrier to the use of culture and susceptibility testing [22, 23], although it remains unclear whether this is a perceived or actual limitation. In one small study exploring pet owners' views on antimicrobial treatments, 20% reported that they would refuse additional diagnostic procedures, such as culture and susceptibility testing, owing to financial concerns [24]. However, it should be emphasised that cytological evaluation could be usefully employed for guiding any therapy, including the use of topical antimicrobials.

Antimicrobials were prescribed for non-antimicrobial effects in 4% of prescriptions. This included the use of ketoconazole and doxycycline for a variety of clinical conditions. Both these drugs have purported anti-inflammatory effects and are used for the treatment of dermatological conditions beyond the treatment of infection [25–27]. However, the reason for selection in this study is unknown. Use of antimicrobial agents for non-antimicrobial effects may have implications for treatment of infection. For example, doxycycline is often recommended for empirical therapy of *Staphylococcus aureus* [17]. Zoonotic azole-resistant yeast infections, such as *Candida auris* and *Malassezia pachydermatis*, are an emerging threat in both humans and dogs [24, 28–30]. Using antimicrobials when not necessary should be avoided to maintain the efficacy of these medications for the treatment of infections.

Overall, prescribing practices in this study were variably consistent with ISCAID superficial bacterial folliculitis guidelines [15, 16]. Most cases of pyoderma received antiseptic therapy, and use of clindamycin was moderate, even though third-generation cephalosporin use was high. The majority of clinics reported use of published guidelines for the treatment of clinical conditions, although only half utilised a formal approach to AS. Barriers reported for implementation of AS in this study are consistent with other surveys, including a lack of staff time and commitment from leadership [31]. Additionally, uncertainty about how to start an AS programme also was reported. Increased awareness of existing resources [32] to aid the creation of AS programmes is needed and may hasten uptake and adherence to new guidelines.

This study has several limitations. First, the study represents dogs and cats presenting to referral dermatology services in the United States in August, and thus dermatological diseases with seasonal fluctuations may be under- or over-represented. Rare conditions or uncommonly prescribed antimicrobials may not be captured. Data about each patient were only collected for one visit, so treatment cannot be correlated to clinical outcomes.

## 5 | Conclusions

This study provides a snapshot of antimicrobial and antiseptic use for cats and dogs presenting to veterinary referral dermatology services. Nevertheless, the use of third-generation cephalosporins was frequent in this population and may not be warranted. Indeed, as mentioned, antimicrobial susceptibility to first- and third-generation cephalosporins is very highly correlated. Clinical outcome studies are needed to determine if third-generation cephalosporins provide an advantage over first-generation cephalosporins; in the absence of such data, first-generation cephalosporins should be prioritised when systemic antibiotic therapy is necessary. According to antimicrobial use guidelines, topical antiseptics are favoured as the mainstay of treatment for surface and superficial pyoderma. Increased awareness of guidelines through continuing education may shift prescribing practices in both referral and primary care settings. As the majority of referral practices in this study indicated using the first iteration of ISCAID guidelines for superficial bacterial folliculitis [15], this study can serve as a benchmark for antimicrobial prescribing in referral dermatology settings and could be repeated in the future to determine adherence to new guideline recommendations.

## Supplementary Material

Supplemental material

Additional supporting information can be found online in the Supporting Information section. Appendix S1: Pre-study survey evaluating clinic’s antimicrobial stewardship and infection prevention and control practices, and clinical approach to select dermatological infections.

## Figures and Tables

**TABLE 1 |:** Systemic and topical antibiotic drugs prescribed by species.

Antibiotic drug name	Canine prescriptions (*n* = 228)	Feline prescriptions (*n* = 16)	Total (*n* = 244)
**Systemic preparation**	**123 (53.9%)**	**11 (68.8%)**	**134 (54.9%)**
Cefpodoxime proxetil	48 (39.0%)	2 (18.2%)	50 (37.3%)
Clindamycin	24 (19.5%)	1 (9.1%)	25 (18.7%)
Cefovecin	6 (4.9%)	5 (45.5%)	11 (8.2%)
Doxycycline	10 (8.1%)	0 (0%)	10 (7.5%)
Cephalexin	9 (7.3%)	0 (0%)	9 (6.7%)
Marbofloxacin	9 (7.3%)	0 (0%)	9 (6.7%)
Amoxicillin–clavulanic acid	2 (1.6%)	2 (18.2%)	4 (3.0%)
Ciprofloxacin	3 (2.4%)	0 (0%)	3 (2.2%)
Enrofloxacin	3 (2.4%)	0 (0%)	3 (2.2%)
Linezolid	3 (2.4%)	0 (0%)	3 (2.2%)
Minocycline	2 (1.6%)	0 (0%)	2 (1.5%)
Rifampin	2 (1.6%)	0 (0%)	2 (1.5%)
Azithromycin	0 (0%)	1 (9.1%)	1 (0.7%)
Chloramphenicol	1 (0.8%)	0 (0%)	1 (0.7%)
Levofloxacin	1 (0.8%)	0 (0%)	1 (0.7%)
**Topical preparation**	**105 (46.1%)**	**5 (31.3%)**	**110 (45.1%)**
Enrofloxacin	31 (29.5%)	3 (60.0%)	34 (30.9%)
Mupirocin	20 (19.0%)	0 (0%)	20 (18.2%)
Silver sulfadiazine	11 (10.5%)	1 (20.0%)	12 (10.9%)
Amikacin	11 (10.5%)	0 (0%)	11 (10.0%)
Gentamicin	5 (4.8%)	0 (0%)	5 (4.5%)
Neomycin–polymyxin B–dexamethasone	5 (4.8%)	0 (0%)	5 (4.5%)
Ceftazidime	4 (3.8%)	0 (0%)	4 (3.6%)
Florfenicol	3 (2.9%)	0 (0%)	3 (2.7%)
Orbifloxacin	3 (2.9%)	0 (0%)	3 (2.7%)
Triple antibiotic	3 (2.9%)	0 (0%)	3 (2.7%)
Gentamicin–betamethasone	2 (1.9%)	0 (0%)	2 (1.8%)
Neomycin	2 (1.9%)	0 (0%)	2 (1.8%)
Polymyxin B	2 (1.9%)	0 (0%)	2 (1.8%)
Tobramycin sulphate	1 (1.0%)	1 (20.0%)	2 (1.8%)
Azithromycin	1 (1.0%)	0 (0%)	1 (0.9%)
Enrofloxacin–dexamethasone	1 (1.0%)	0 (0%)	1 (0.9%)

*Note:* Bold indicates that the rows below “Systemic preparations” (which represent the total of all systemic preparations) are all systemic drugs, and the rows below “Topical preparation” are all topical drug formulations.

**TABLE 2 |:** Systemic and topical antifungal drugs prescribed by species.

Antifungal drug name	Canine prescriptions (*n* = 117)	Feline prescriptions (*n* = 5)	Total (*n* = 122)
**Systemic preparation**	**72 (61.5%)**	**1 (20.0%)**	**73 (59.8%)**
Ketoconazole	40 (55.6%)	0 (0%)	40 (54.8%)
Fluconazole	22 (30.6%)	1 (100%)	23 (31.5%)
Terbinafine	7 (9.7%)	0 (0%)	7 (9.6%)
Itraconazole	3 (4.2%)	0 (0%)	3 (4.1%)
**Topical preparation**	**45 (38.5%)**	**4 (80.0%)**	**49 (40.2%)**
Miconazole	27 (60.0%)	2 (50.0%)	29 (59.2%)
Clotrimazole	5 (11.1%)	1 (25.0%)	6 (12.2%)
Posaconazole	3 (6.7%)	1 (25.0%)	4 (8.2%)
Ketoconazole	3 (6.7%)	0 (0%)	3 (6.1%)
Miconazole–dexamethasone	3 (6.7%)	0 (0%)	3 (6.1%)
Clotrimazole–dexamethasone	2 (4.4%)	0 (0%)	2 (4.1%)
Terbinafine	2 (4.4%)	0 (0%)	2 (4.1%)

**TABLE 3 |:** Topical antibiotic–antifungal combination drugs prescribed by species.

Antibiotic–antifungal combination drug name	Canine prescriptions (*n* = 73)	Feline prescriptions (*n* = 4)	Total (*n* = 77)
Gentamicin–clotrimazole–betamethasone	15 (20.5%)	1 (25.0%)	16 (20.8%)
Florfenicol–terbinafine–mometasone	13 (17.8%)	0 (0%)	13 (16.9%)
Gentamicin–clotrimazole–mometasone	8 (11.0%)	0 (0%)	8 (10.4%)
Gentamicin–miconazole–hydrocortisone aceponate	8 (11.0%)	0 (0%)	8 (10.4%)
Neomycin–thiostrepton–nystatin–triamcinolone	8 (11.0%)	0 (0%)	8 (10.4%)
Orbifloxacin–posaconazole–mometasone	7 (9.6%)	1 (25.0%)	8 (10.4%)
Enrofloxacin–miconazole	6 (8.2%)	0 (0%)	6 (7.8%)
Polymyxin B–miconazole–prednisolone	5 (6.8%)	1 (25.0%)	6 (7.8%)
Florfenicol–terbinafine–beta-methasone	1 (1.4%)	1 (25.0%)	2 (2.6%)
Enrofloxacin–miconazole–dexamethasone	1 (1.4%)	0 (0%)	1 (1.3%)
Mupirocin–ketoconazole	1 (1.4%)	0 (0%)	1 (1.3%)

**TABLE 4 |:** Antiseptics prescribed by species.

Antiseptic drug name	Canine prescriptions (*n* = 482)	Feline prescriptions (*n* = 19)	Total (*n* = 501)
Chlorhexidine	147 (30.5%)	5 (26.3%)	152 (30.3%)
Chlorhexidine–miconazole–TrizEDTA	45 (9.3%)	2 (10.5%)	47 (9.4%)
Chlorhexidine–miconazole	45 (9.3%)	0 (0%)	45 (9.0%)
TrizEDTA	41 (8.5%)	0 (0%)	41 (8.2%)
Chlorhexidine–ketoconazole	29 (6.0%)	2 (10.5%)	31 (6.2%)
TrizEDTA–ketoconazole	21 (4.4%)	3 (15.8%)	24 (4.8%)
Salicylic acid	22 (4.6%)	1 (5.3%)	23 (4.6%)
Salicylic acid–chloroxylenol–EDTA	16 (3.3%)	1 (5.3%)	17 (3.4%)
Acetic acid–boric acid–ketoconazole–hydrocortisone (e.g., Malacetic Ultra)	11 (2.3%)	1 (5.3%)	12 (2.4%)
Chloroxylenol	10 (2.1%)	2 (10.5%)	12 (2.4%)
TrizEDTA–chlorhexidine	12 (2.5%)	0 (0%)	12 (2.4%)
Acetic acid–boric acid (e.g., Malacetic)	11 (2.3%)	0 (0%)	11 (2.2%)
Aluminium acetate	9 (1.9%)	0 (0%)	9 (1.8%)
Hypochlorous acid	8 (1.7%)	0 (0%)	8 (1.6%)
Sodium hypochlorite	7 (1.5%)	0 (0%)	7 (1.4%)
Selenium sulphide	6 (1.2%)	0 (0%)	6 (1.2%)
Benzoyl peroxide	5 (1.0%)	0 (0%)	5 (1.0%)
Phytosphingosine	5 (1.0%)	0 (0%)	5 (1.0%)
Zymox	4 (0.8%)	1 (5.3%)	5 (1.0%)
Chlorhexidine–miconazole–microsilver	4 (0.8%)	0 (0%)	4 (0.8%)
Chlorhexidine–climbazole	3 (0.6%)	0 (0%)	3 (0.6%)
Ketoconazole	3 (0.6%)	0 (0%)	3 (0.6%)
Ophytrium	3 (0.6%)	0 (0%)	3 (0.6%)
Silver	3 (0.6%)	0 (0%)	3 (0.6%)
DisodiumEDTA–salicylic acid	1 (0.2%)	1 (5.3%)	2 (0.4%)
Benzoyl peroxide–salicylic acid	1 (0.2%)	0 (0%)	1 (0.2%)
Benzyl alcohol	1 (0.2%)	0 (0%)	1 (0.2%)
Chlorhexidine–ophytrium	1 (0.2%)	0 (0%)	1 (0.2%)
Chloroxylenol–miconazole	1 (0.2%)	0 (0%)	1 (0.2%)
Hydrogen peroxide	1 (0.2%)	0 (0%)	1 (0.2%)
Hydroxyl acids (e.g., Acetic, Boric, Lactic, Malic acids)	1 (0.2%)	0 (0%)	1 (0.2%)
Miconazole	1 (0.2%)	0 (0%)	1 (0.2%)
Miconazole–dexamethasone	1 (0.2%)	0 (0%)	1 (0.2%)
Not recorded in medical record	1 (0.2%)	0 (0%)	1 (0.2%)
Tinactin	1 (0.2%)	0 (0%)	1 (0.2%)
TrizEDTA/*N*-Acetylcysteine	1 (0.2%)	0 (0%)	1 (0.2%)

**TABLE 5 |:** Categories of clinical conditions and frequency of antimicrobial prescribing.

Clinical condition categories	Canine (*n* = 359/762)	Feline (*n* = 21/83)	Total (*n* = 380/845)
Skin	173/429 (40.3%)	7/44 (15.9%)	180/473 (38.1%)
Otic (ear)	136/211 (64.5%)	12/31 (38.7%)	148/242 (61.2%)
Interdigital	32/77 (41.6%)	0/2 (0%)	32/79 (40.5%)
Ear/pinnae	5/13 (38.5%)	0/1 (0%)	5/14 (35.7%)
Perianal/perineal	4/10 (40.0%)	1/1 (100%)	5/11 (45.5%)
Nasal planum	3/9 (33.3%)	1/2 (50.0%)	4/11 (36.4%)
Nailbed	2/8 (25.0%)	0/1 (0%)	2/9 (22.2%)
Mucocutaneous junctions	4/5 (80.0%)	0/1 (0%)	4/6 (66.7%)

**TABLE 6 |:** Number of dogs prescribed antimicrobials and antiseptics for skin pyoderma problems.

Dogs only	Deep pyoderma (*n* = 16)	Superficial focal pyoderma (*n* = 25)	Superficial generalised pyoderma (*n* = 46)	Surface pyoderma (*n* = 7)	Cutaneous dysbiosis/bacterial or yeast overgrowth (*n* = 2)	Cellulitis (*n* = 2)
No prescriptions	0 (0%)	2 (8.0%)	1 (2.2%)	0 (0%)	0 (0%)	1 (50.0%)
Antiseptic	13 (81.3%)	19 (76.0%)	43 (93.5%)	7 (100%)	1 (50.0%)	0 (0%)
Topical antimicrobial	7 (43.8%)	8 (32.0%)	2 (4.3%)	1 (14.3%)	1 (50.0%)	0 (0%)
Systemic antimicrobial	14 (87.5%)	3 (12.0%)	23 (50.0%)	3 (42.9%)	1 (50.0%)	1 (50.0%)

## Data Availability

The data that support the findings of this study are available from the corresponding author upon reasonable request.
