# Supplementary material for: Prevalence of Antimicrobial Use in Small Animal Dermatology Referral Practices in the United States
Source: Vet Dermatol. 2026 May 27;37(5):581–91. doi: 10.1111/vde.70087 (PMC13318191; doi:10.1111/vde.70087)
Supplement: Supplementary file 1 [file VDE-37-581-s001.pdf]

APPENDIX S1. Pre-study survey evaluating clinic's antimicrobial stewardship and infection prevention and control practices, and clinical approach to select dermatological infections

2023 Antibiotic use in dogs and cats study: facility survey for U.S. dermatological referral practices

Thank you for completing this survey and for participating in the 2023 U.S. Dermatology Referral Practices Antibiotic Use Study in Dogs and Cats. This survey includes general information about your clinic and should take approximately 10 min to complete. It should be completed by an employee who has a working knowledge of your clinic and awareness of any antibiotic stewardship and infection prevention initiatives in place. **Please have only one person from your clinic complete the survey, and please try and complete it in one sitting.**

Questions should be directed to the XXX researchers at XXX.

Please keep the following definitions in mind when completing the survey:

- <u>Small animal</u>: refers to only dogs and cats.
- <u>Antimicrobial resistance</u>: the ability of micro-organisms (including bacteria) to resist the effects of antimicrobials (including antibiotics).
- <u>Antimicrobial stewardship</u>: the process of improving how we use antimicrobial drugs (including antibiotic drugs) while effectively treating infections.
- <u>Antimicrobial use</u>: any time an antimicrobial (including antibiotics) is used in veterinary medicine, regardless of the prescriber's intention (e.g. infection treatment or prevention, stimulation of GI motility, disease control in a group of animals).

Background Information

1. What is the clinic's name for which you are completing this survey?
   a. _______

2. In what U.S. state is [clinic_name]'s located?
   a. __drop down of 50 states, plus Washington DC_____
   b. Other: ________

3. What is [clinic_name]'s 5-digit zip code?
   a. __________

4. In what county is [clinic_name] located?
   a. __________

5. What type of clinic is [clinic_name]?
   A nonacademic referral hospital is defined as a veterinary practice that offers specialised services and/or diagnostic testing.

A veterinary teaching hospital is defined as an accredited academic university that has a veterinary teaching hospital and DVM curriculum.

a. General practice/primary care clinic
b. Nonacademic referral hospital
c. Shelter setting
d. Veterinary teaching hospital (accredited academic university)

6. How many board-certified referral clinicians are on staff (full-time or part-time) at [clinic_name]? *(whole number only)*
   a. ______

Antimicrobial Prescribing

7. What systemic antibiotic is typically prescribed as empirical choice for a cutaneous dysbiosis (bacterial overgrowth) in dogs?
   a. Amoxicillin-clavulanic acid
   b. Cephalexin
   c. Clindamycin
   d. Doxycycline
   e. Fluoroquinolones
   f. Third-generation cephalosporins (e.g. Convenia, Simplicef)
   g. Trimethoprim-sulfamethoxazole (TMS)
   h. None; I generally start with topical antimicrobials
   i. None; generally start with topical or systemic glucocorticoids/anti-inflammatory medications
   j. None; I would not prescribe any antimicrobial (systemic or topical) therapy or systemic glucocorticoids/anti-inflammatory medications

8. What systemic antibiotic is typically prescribed as empirical choice for a staphylococcal surface pyoderma in dogs?
   a. Amoxicillin-clavulanic acid
   b. Cephalexin
   c. Clindamycin
   d. Doxycycline
   e. Fluoroquinolones
   f. Third-generation cephalosporins (e.g. Convenia, Simplicef)
   g. Trimethoprim-sulfamethoxazole (TMS)
   h. None; I generally start with topical antimicrobials
   i. None; I would not prescribe an antibiotic (systemic or topical)

9. What systemic antibiotic is typically prescribed as empirical choice for a staphylococcal superficial pyoderma in dogs?
   a. Amoxicillin-clavulanic acid
   b. Cephalexin
   c. Clindamycin
   d. Doxycycline
   e. Fluoroquinolones

    f. Third-generation cephalosporins (e.g. Convenia, Simplicef)
    g. Trimethoprim-sulfamethoxazole (TMS)
    h. None; I generally start with topical antimicrobials
    i. None; I would not prescribe an antibiotic (systemic or topical)

10. What systemic antibiotic is typically prescribed as empirical choice for a staphylococcal deep pyoderma in dogs?
    a. Amoxicillin-clavulanic acid
    b. Cephalexin
    c. Clindamycin
    d. Doxycycline
    e. Fluoroquinolones
    f. Third-generation cephalosporins (e.g. Convenia, Simplicef)
    g. Trimethoprim-sulfamethoxazole (TMS)
    h. None; I generally perform culture before starting an antibiotic
    i. None; I generally start with topical antibiotics
    j. None; I would not prescribe an antibiotic (systemic or topical)

11. What systemic antibiotic is typically prescribed as empirical choice for a superficial pyoderma associated with rod-shaped bacteria in dogs?
    a. Amoxicillin-clavulanic acid
    b. Clindamycin
    c. Fluoroquinolones
    d. Third-generation cephalosporins (e.g. Convenia, Simplicef)
    e. Trimethoprim-sulfamethoxazole (TMS)
    f. None; I generally perform culture before starting an antibiotic
    g. None; I generally start with topical antibiotics
    h. None; I would not prescribe an antibiotic (systemic or topical)

12. What systemic antibiotic is typically prescribed as empirical choice for a deep pyoderma associated with rod-shape bacteria in dogs?
    a. Amoxicillin-clavulanic acid
    b. Clindamycin
    c. Fluoroquinolones
    d. Third-generation cephalosporins (e.g. Convenia, Simplicef)
    e. Trimethoprim-sulfamethoxazole (TMS)
    f. None; I generally perform culture before starting an antibiotic
    g. None; I generally start with topical antibiotics
    h. None; I would not prescribe an antibiotic (systemic or topical)

13. A dog is brought to you for a dermatological issue. The owner has already started him on antibiotic based or previous experience, without veterinary surgeon guidance. Upon examination you deem the antibiotic is not necessary. How do you respond to the client's actions? *(select all that apply)*
    a. Ignore the client's actions of initiating antibiotic therapy

b. Instruct the client to continue treatment with the antibiotic for an additional 7 days
c. Tell the client to discontinue antibiotic treatment
d. Discuss with the client why antibiotics are not indicated in this case
e. Recommend that the client not initiate antibiotic therapy unless instructed by a veterinary surgeon
f. Discuss with the client potential harms of unnecessary antibiotic use
g. Other: __________
h. None of the above

Antimicrobial resistance and antimicrobial stewardship

14. Does [clinic_name] utilise published small animal antibiotic use guidelines for specific conditions (e.g. International Society for Companion Animal Infectious Diseases guideline for superficial bacterial folliculitis in dogs)?
    a. Yes
    b. No
    c. Unsure

15. Does [clinic_name] take a formal approach (e.g. established programme, protocols, policies) to antimicrobial stewardship?
    a. Yes
    b. No
    c. Unsure

16. Does [clinic_name] have an antimicrobial stewardship committee?
    a. Yes
        i. What roles are represented on the antimicrobial stewardship committee? *(select all that apply)*
            1. Veterinary surgeon(s) – small animal
            2. Veterinary surgeon(s) – large animal
            3. Veterinary technician/nurse(s)
            4. Veterinary assistant(s)
            5. Veterinary student(s)
            6. Infection preventionist(s)
            7. Microbiology technician(s)
            8. Veterinary microbiologist(s)
            9. Pharmacologist(s)
            10. Practice manager(s)
            11. Hospital director(s)
            12. Pharmacist(s)
            13. Veterinary public health faculty/staff
            14. Office administrator(s) or receptionist(s)
            15. Other: _________
    b. No
        ii. Has there been interest in establishing an antimicrobial stewardship committee?

1. Yes
2. No
3. Unsure

c. Unsure
   a. Has there been interest in establishing an antimicrobial stewardship committee?
      i. Yes
      ii. No
      iii. Unsure

17. What are or have been the major barriers to taking action regarding antimicrobial stewardship at [clinic_name]? *(select all that apply)*
    a. Lack of formal commitment or interest from clinic *leadership*
    b. Lack of formal commitment or interest from clinic *staff*
    c. Lack of staff time dedicated to antimicrobial stewardship activities
    d. Lack of dedicated resources (e.g. money) to antimicrobial stewardship activities
    e. Lack of awareness of the importance of an antimicrobial stewardship committee
    f. Lack of training regarding antimicrobial stewardship practices and initiatives
    g. Uncertainty of how to initiate establishment of an antimicrobial stewardship committee
    h. Other: _____________
    i. None

18. Does [clinic_name] take a formal approach (e.g. established programme, protocols, policies) to infection prevention and control?
    a. Yes
    b. No
    c. Unsure

19. Does [clinic_name] have an infection control programme?
    The goals of an infection control programme include prevention of healthcare-associated infections and transmission of pathogens between patients.
    a. Yes
       i. Is this the same committee as your antimicrobial stewardship committee?
          1. Yes
             a. Do you have separate meetings for infection control and antimicrobial stewardship focuses/topics?
                i. Yes
                ii. No
          2. No
             a. What roles are represented on the infection control programme? *(select all that apply)*

i. Veterinary surgeon(s) – small animal
ii. Veterinary surgeon(s) – large animal
iii. Veterinary technician/nurse(s)
iv. Veterinary assistant(s)
v. Veterinary student(s)
vi. Infection preventionist(s)
vii. Microbiology technician(s)
viii. Veterinary microbiologist(s)
ix. Pharmacologist(s)
x. Practice manager(s)
xi. Hospital director(s)
xii. Pharmacist(s)
xiii. Veterinary public health faculty/staff
xiv. Office administrator(s) or receptionist(s)
xv. Other: _________

b. No
    i. Has there been interest in establishing an infection control programme?
        1. Yes
        2. No
        3. Unsure
c. Unsure
    i. Has there been interest in establishing an infection control programme?
        1. Yes
        2. No
        3. Unsure

20. What are or have been the major barriers to taking action regarding infection control at [clinic_name]? *(select all that apply)*
    a. Lack of faculty or staff with infection prevention expertise
    b. Lack of formal commitment or interest from clinic leadership
    c. Lack of formal commitment or interest from clinic staff
    d. Lack of staff time dedicated to infection prevention activities
    e. Lack of dedicated resources (e.g. money) to infection prevention activities
    f. Lack of awareness of the importance of infection prevention
    g. Uncertainty of how to run an infection control programme
    h. Other: _____________
    i. None

21. Does [clinic_name] require any training specific to antimicrobial stewardship? *(select all that apply)*
    a. Antimicrobial stewardship continuing education/training for *clinicians*
    b. Antimicrobial stewardship continuing education/training for *veterinary technicians/nurses and support staff*
    c. Other: ____________

    d. None

22. What factor(s) are most important to address in regards to optimising antibiotic use in veterinary dermatology clinics? *(select all that apply)*
    a. Alternatives to antibiotic use for common conditions
    b. Client communication support
    c. Duration of antibiotic use
    d. Specific antibiotic selection recommendations for common conditions
    e. Other: ______
    f. None

23. What administrative actions have clinic leadership taken at [clinic_name]? *(select all that apply)*
    a. Dedicated funds for antimicrobial stewardship initiatives
    b. Dedicated salary funding for antimicrobial stewardship staff time
    c. Dedicated funds for infection prevention activities
    d. Dedicated salary funding for infection prevention staff time
    e. Developed facility-specific prescribing protocols based on published guidelines
    f. Development of an antimicrobial stewardship plan or policy
    g. Identified a veterinary leader for antimicrobial stewardship initiatives
    h. Provided formal communication from clinic administration to clinicians, veterinary technicians/nurses, and support staff about their commitment to antimicrobial stewardship
    i. Other: ____________
    j. None

24. What antimicrobial stewardship interventions have been implemented at [clinic_name]? *(select all that apply)*
    a. Established protocols for submission of bacterial and fungal culture and susceptibility testing
    b. Identified clinical conditions to target for improved prescribing
    c. Mandatory inclusion of diagnosis and/or indication for antimicrobial prescriptions in medical record
    d. Measure and/or evaluate antimicrobial prescribing practices
        i. In which of the following ways does [clinic_name] measure and/or evaluate antimicrobial prescribing practices? *(select all that apply)*
            a. Audit and feedback to summarise and inform clinicians about their prescribing behaviours
            b. Measure and track prescribing of specific antibiotics (e.g. cefovecin, enrofloxacin)
            c. Measure and track syndrome-specific prescribing (e.g. pets presenting for pyoderma)
            d. Other: __________
            e. None
    e. Pre-authorisation requirement for certain antibiotics

f. Regular review of inpatient antibiotics 48–72 h after first administration (i.e. “antibiotic time-out”)
g. Systemically assessed outcomes of prescribed antimicrobial drug therapy
h. Use of clinic-specific antibiogram
i. Other: ____________
j. None

25. Are any of the following client communication tools used clinic-wide in [clinic_name]? *(select all that apply)*
    a. Client fact sheets about antimicrobials and/or antimicrobial resistance
    b. Client information on antibiotic alternatives (e.g. diet change, medicated shampoos)
    c. Disease prevention protocols for common clinical conditions (e.g. atopy)
    d. Non-antibiotic prescription pad (e.g. https://arsi.umn.edu/noabx)
    e. Visible clinic commitment (e.g. poster, pamphlet) to responsible antimicrobial use
    f. Other: ____________
    g. None

26. What kinds of support would help in the implementation and/or improvement of antimicrobial stewardship activities at [clinic_name]? *(select all that apply)*
    a. Client education on antimicrobial resistance and the importance of using antimicrobials only when needed
    b. Materials to guide engagement with clinic leadership
    c. Prescribing guidelines specific to common small animal syndromes
    d. Sample antimicrobial stewardship plan or policy for small animal dermatology clinics
    e. Clinical treatment consultation for difficult cases
    f. Collaboration with other local clinics/hospitals to share and implement best practices
    g. Dedicated funds for antimicrobial stewardship programmes
    h. Dedicated salary funding for staff antimicrobial stewardship effort
    i. Electronic health records software/technology support
    j. Formal commitment from clinic leadership
    k. Inclusion of clinic antimicrobial stewardship responsibilities in staff job descriptions
    l. Continuing education opportunities for antimicrobial stewardship in small animal medicine
    m. Listserv to provide education emails and event updates
    n. State or federal regulations requiring clinic-based antimicrobial stewardship programmes
    o. State or national antimicrobial stewardship conference for clinicians specific to small animal medicine
    p. Other: _________
    q. None

27. Please share any other information you would like to about [clinic_name]'s antimicrobial stewardship practices.
    a. _______________

Thank you for taking the time to complete this survey and for your active involvement in the U.S. Dermatology Referral Practices Antibiotic Use Study in Dogs and Cats. Your dedication to research will help advance antibiotic stewardship across the U.S.

Please visit https://arsi.umn.edu for free antimicrobial stewardship resources.

APPENDIX S2. List of diagnostic tests

Culture and susceptibility – bacterial (not urine)
Culture and susceptibility – bacterial (urine)
Culture – fungal
Culture – mycobacterial
Cytological investigation (select all that apply)
- Cocci
- Dimorphic fungal organisms
- Inflammatory cells – lymphocytes
- Inflammatory cells – macrophages
- Inflammatory cells – neutrophils
- Neoplastic cells
- Rods
- Yeast – *Malassezia*
- No significant findings

Histopathological investigation
PCR – fungal
PCR – *Leishmania*
PCR – Mycobacterial
PCR – Oomycetes
Serology/titres – fungal
Serology/titres – *Leishmania*
Serology/titres – *Pythium*
Urinalysis

TABLE S1. Criteria for determining the level of evidence of infection.

Evidence of infection	Criteria
Confirmed infection	Documentation of any of the following: 1) positive culture; 2) cytological/fluid analysis with presence of organisms with clinical signs of infection at the site of collection; 3) positive PCR with clinical signs of disease; 4) 4-fold rise in serological titre.
Suspected infection	Documentation of any of the following: 1) wound (surgical or open) with fever or redness or tenderness or warmth or swelling or bite history; 2) neutrophilic fluid/cytological findings with no organisms seen; 3) single positive serology with clinical signs of disease; 4) radiographs identifying pneumonia yet without positive airway wash and/or C&S; 5) purulent skin disease without cytology or C&S; 6) purulent discharge from an orifice without cytological results or C&S; 7) visualisation of gastrointestinal perforation in the absence of ”confirmed infection”; 8) fever of unknown origin; 9) fever with indwelling device (e.g. urinary catheter, central line, implant with evidence of infection at the implant site); 10) lytic bony lesion; 11) echocardiographic evidence of vegetative lesion on heart valve.
No evidence of infection	No documentation of confirmed/suspected infection or if an alternative reason for antimicrobial. Includes documented negative titres or cultures, no titres or cultures submitted, ”preventative” uses, as written in medical record, or systemic antimicrobial use after clean surgery. Alternative noninfectious diagnosis that explains clinical signs.

C&S, culture and susceptibility testing

TABLE S2. Canine and feline demographics

	Dogs n = 479 (%)	Cats n = 71 (%)	Total count n = 550 (%)
**Species**			
Dog	--	--	479 (87.1%)
Cat	--	--	71 (12.9%)
**Sex**			
Male neutered	222 (46.3%)	33 (46.5%)	255 (46.4%)
Male intact	43 (9.0%)	1 (1.4%)	44 (8.0%)
Female spayed	192 (40.1%)	37 (52.1%)	229 (41.6%)
Female intact	22 (4.6%)	0 (0%)	22 (4.0%)
**Age**			
< 4 months	1 (0.2%)	0 (0%)	1 (0.2%)
> 4–12 months	5 (1.3%)	2 (2.8%)	8 (1.5%)
> 1– 3 years	69 (14.4%)	8 (11.3%)	77 (14.0%)
> 3– 7 years	207 (43.2%)	20 (28.2%)	227 (41.3%)
> 7– 10 years	101 (21.1%)	16 (22.5%)	117 (21.3%)
> 10– 15 years	90 (18.8%)	22 (31.0%)	112 (20.4%)
> 15– 20 years	5 (1.0%)	3 (4.2%)	8 (1.5%)
Average (years)	6.6	7.9	6.8
Median (years)	6.0	7.9	6.2
**Comorbidity**			
Yes	54 (11.3%)	4 (5.6%)	58 (10.5%)
Cushing's disease	11 (20.4%)	0 (0%)	11 (19.0%)
Dental disease	10 (18.5%)	2 (50.0%)	12 (20.7%)
Diabetes mellitus	2 (3.7%)	1 (25.0%)	3 (5.2%)
Hypothyroidism	13 (24.1%)	0 (0%)	13 (22.4%)
Inflammatory bowel disease/chronic enteropathy	7 (13.0%)	0 (0%)	7 (12.1%)
Neoplasia	18 (33.3%)	1 (25.0%)	19 (32.8%)
No	420 (87.7%)	66 (93.0%)	486 (88.4%)
Not recorded	5 (1.0%)	1 (1.4%)	6 (1.1%)
**Immunocompromising drugs**			
Yes	289 (60.3%)	25 (35.2%)	314 (57.1%)
No	190 (39.7%)	46 (64.8%)	236 (42.9%)
**Weight**			
Mean (kg)	24.0	5.2	21.6
Median (kg)	23.8	5.3	20.4
Range	2–93	2.3–8.6	2–93
Missing	1	0	1
**Region**			
South	179 (37.4%)	21 (29.6%)	200 (36.4%)
Midwest	108 (22.5%)	17 (23.9%)	125 (22.7%)
Northeast	108 (22.5%)	17 (23.9%)	125 (22.7%)

West	84 (17.5%)	16 (22.5%)	100 (18.2%)
**Clinic type**			
Accredited Teaching Hospital	170 (35.5%)	30 (42.3%)	200 (36.4%)
Nonacademic referral	309 (64.5%)	41 (57.7%)	350 (63.6%)

TABLE S3. Clinical conditions by species

Clinical conditions	Canine (n = 762)	Feline (n = 83)	Total (n = 845)
Cutaneous	429 (56.3%)	44 (53.0%)	473 (56.0%)
• Dermatitis	• 247 (57.6%)	• 27 (61.4%)	• 274 (57.9%)
○ Atopic (environmental allergy)	○ 138 (32.2%)	○ 14 (31.8%)	○ 152 (32.1%)
○ Allergy of unknown aetiology	○ 67 (15.6%)	○ 11 (25.0%)	○ 78 (16.5%)
○ *Malassezia*	○ 22 (5.1%)	○ 1 (2.3%)	○ 23 (4.9%)
○ Food allergy	○ 16 (3.7%)	○ 1 (2.3%)	○ 17 (3.6%)
○ Flea allergy	○ 4 (0.9%)	○ 0 (0%)	○ 4 (0.8%)
• Pyoderma	• 98 (22.8%)	• 2 (4.5%)	• 100 (21.1%)
○ Superficial generalised	○ 46 (10.7%)	○ 1 (2.3%)	○ 47 (9.9%)
○ Superficial focal	○ 25 (5.8%)	○ 0 (0%)	○ 25 (5.3%)
○ Deep	○ 16 (3.7%)	○ 0 (0%)	○ 16 (3.4%)
○ Surface	○ 7 (1.6%)	○ 1 (2.3%)	○ 8 (1.7%)
○ Cellulitis	○ 2 (0.5%)	○ 0 (0%)	○ 2 (0.4%)
○ Cutaneous dysbiosis/bacterial or yeast overgrowth	○ 2 (0.5%)	○ 0 (0%)	○ 2 (0.4%)
• Other	• 26 (6.1%)	• 7 (15.9%)	• 33 (7.0%)
• Open diagnosis	• 16 (3.7%)	• 2 (4.5%)	• 18 (3.8%)
• Pemphigus foliaceus	• 11 (2.6%)	• 3 (6.8%)	• 14 (3.0%)
• Cutaneous neoplasia	• 10 (2.3%)	• 0 (0%)	• 10 (2.1%)
• Sebaceous adenitis	• 5 (1.2%)	• 0 (0%)	• 5 (1.1%)
• Dermatophytosis	• 2 (4.7%)	• 2 (4.5%)	• 4 (0.8%)
• Cushing’s disease	• 3 (0.7%)	• 0 (0%)	• 3 (0.6%)
• Demodicosis	• 3 (0.7%)	• 0 (0%)	• 3 (0.6%)
• Alopecia X	• 2 (0.5%)	• 0 (0%)	• 2 (0.4%)
• Abscess	• 1 (0.2%)	• 0 (0%)	• 1 (0.2%)
• Cutaneous lupus erythematosus	• 0 (0%)	• 1 (2.3%)	• 1 (0.2%)
• Hair follicle dysplasia	• 1 (0.2%)	• 0 (0%)	• 1 (0.2%)
• Sterile nodular panniculitis	• 1 (0.2%)	• 0 (0%)	• 1 (0.2%)
• Systemic fungal infection	• 1 (0.2%)	• 0 (0%)	• 1 (0.2%)
• Wound – bite	• 1 (0.2%)	• 0 (0%)	• 1 (0.2%)
• Wound – traumatic	• 1 (0.2%)	• 0 (0%)	• 1 (0.2%)
Otic (Ear)	211 (27.7%)	31 (37.3%)	242 (28.6%)
• Otitis externa	• 175 (82.9%)	• 15 (48.4%)	• 190 (78.5%)
○ Caused by allergy of unknown aetiology	○ 66 (37.7%)	○ 0 (0%)	○ 66 (34.7%)
○ Caused by atopic dermatitis (environmental allergy)	○ 53 (30.3%)	○ 2 (13.3%)	○ 55 (28.9%)
○ Caused by autoimmune condition (e.g. pemphigus foliaceus)	○ 1 (0.6%)	○ 0 (0%)	○ 1 (0.5%)
○ Caused by bacteria	○ 65 (37.1%)	○ 10 (66.7%)	○ 75 (39.5%)
○ Caused by food allergy	○ 13 (7.4%)	○ 0 (0%)	○ 13 (6.8%)

○ Caused by yeast<br>○ No bacteria, parasites or yeast detected<br>○ Other<br>○ Open diagnosis<br>• Otitis media<br>• Other<br>• Open diagnosis<br>• Otitis interna<br>• Not recorded in medical record	○ 58 (33.1%)<br>○ 3 (1.7%)<br>○ 3 (1.7%)<br>○ 1 (0.6%)<br>• 14 (6.6%)<br>• 8 (3.8%)<br>• 10 (4.7%)<br>• 1 (0.5%)<br>• 3 (1.4%)	○ 2 (13.3%)<br>○ 0 (0%)<br>○ 4 (26.7%)<br>○ 1 (6.7%)<br>• 6 (19.4%)<br>• 4 (12.9%)<br>• 1 (3.2%)<br>• 5 (16.1%)<br>• 0 (0%)	○ 60 (31.6%)<br>○ 3 (1.6%)<br>○ 7 (3.7%)<br>○ 2 (1.1%)<br>• 20 (8.3%)<br>• 12 (5.0%)<br>• 11 (4.5%)<br>• 6 (2.5%)<br>• 3 (1.2%)
Interdigital<br>• Dermatitis<br>○ Atopic (environmental allergy)<br>○ *Malassezia*<br>• Dermatitis - allergy of unknown aetiology<br>• Pyoderma<br>• Interdigital furunculosis/cysts<br>• Other<br>• Dermatitis - food allergy<br>• Not recorded in medical record<br>• Open diagnosis	77 (10.1%)<br>• 34 (44.2%)<br>○ 18 (23.4%)<br>○ 16 (20.8%)<br>• 15 (19.5%)<br>• 10 (13.0%)<br>• 8 (10.4%)<br>• 6 (7.8%)<br>• 2 (2.6%)<br>• 1 (1.3%)<br>• 1 (1.3%)	2 (2.4%)<br>• 0 (0%)<br>○ 0 (0%)<br>○ 0 (0%)<br>• 0 (0%)<br>• 0 (0%)<br>• 0 (0%)<br>• 2 (100%)<br>• 0 (0%)<br>• 0 (0%)<br>• 0 (0%)	79 (9.3%)<br>• 34 (43.0%)<br>○ 18 (22.8%)<br>○ 16 (20.3%)<br>• 15 (19.0%)<br>• 10 (12.7%)<br>• 8 (10.1%)<br>• 8 (10.1%)<br>• 2 (2.5%)<br>• 1 (1.3%)<br>• 1 (1.3%)
Ear pinnae<br>• Dermatitis – atopic (environmental allergy)<br>• Dermatitis – allergy of unknown aetiology<br>• Open diagnosis<br>• Vasculitis<br>• Other	13 (1.7%)<br>• 6 (46.2%)<br>• 2 (15.4%)<br>• 2 (15.4%)<br>• 2 (15.4%)<br>• 1 (7.7%)	1 (1.2%)<br>• 0 (0%)<br>• 1 (100%)<br>• 0 (0%)<br>• 0 (0%)<br>• 0 (0%)	14 (1.7%)<br>• 6 (42.9%)<br>• 3 (21.4%)<br>• 2 (14.3%)<br>• 2 (14.3%)<br>• 1 (7.1%)
Perianal/perineal<br>• Perianal fistula<br>• Anal sacculitis<br>• Dermatitis – allergy of unknown aetiology<br>• Dermatitis – *Malassezia*	10 (1.3%)<br>• 5 (50.0%)<br>• 3 (30.0%)<br>• 1 (10.0%)<br>• 1 (10.0%)	1 (1.2%)<br>• 0 (0%)<br>• 0 (0%)<br>• 1 (100%)<br>• 0 (0%)	11 (1.3%)<br>• 5 (45.5%)<br>• 3 (27.3%)<br>• 2 (18.2%)<br>• 1 (9.1%)
Nasal planum<br>• Other<br>• Discoid lupus erythematosus<br>• Nasal hyperkeratosis<br>• Open diagnosis<br>• Vasculitis	9 (1.2%)<br>• 3 (33.3%)<br>• 3 (33.3%)<br>• 1 (11.1%)<br>• 1 (11.1%)<br>• 1 (11.1%)	2 (2.4%)<br>• 2 (100%)<br>• 0 (0%)<br>• 0 (0%)<br>• 0 (0%)<br>• 0 (0%)	11 (1.3%)<br>• 5 (45.5%)<br>• 3 (27.3%)<br>• 1 (9.1%)<br>• 1 (9.1%)<br>• 1 (9.1%)
Nailbed<br>• Bacterial onychitis<br>• Malassezia paronychia	8 (1.0%)<br>• 3 (37.5%)<br>• 3 (37.5%)	1 (1.2%)<br>• 0 (0%)<br>• 0 (0%)	9 (1.1%)<br>• 3 (33.3%)<br>• 3 (33.3%)

• Lupoid onychitis/lupoid onychodystrophy<br>• Pemphigus foliaceus	• 2 (25.0%)<br>• 0 (0%)	• 0 (0%)<br>• 1 (100%)	• 2 (22.2%)<br>• 1 (11.1%)
Mucocutaneous junctions<br>• Mucocutaneous pyoderma<br>• Other<br>• Open diagnosis	5 (0.7%)<br>• 3 (60.0%)<br>• 1 (20.0%)<br>• 1 (20.0%)	1 (1.2%)<br>• 0 (0%)<br>• 1 (100%)<br>• 0 (0%)	6 (0.7%)<br>• 3 (50.0%)<br>• 2 (33.3%)<br>• 1 (16.7%)

TABLE S4. Clinical conditions prescribed at least one antimicrobial

Clinical conditions	Canine (n = 359/762)	Feline (n = 21/83)	Total (n = 380/845)
**Skin**	173/429 (40.3%)	7/44 (15.9%)	180/473 (38.1%)
• Dermatitis – atopic (environmental allergy)	• 46/138 (33.3%)	• 2/14 (14.3%)	• 48/152 (31.6%)
• Dermatitis – allergy of unknown aetiology	• 26/67 (38.8%)	• 2/11 (18.2%)	• 28/78 (35.9%)
• Pyoderma – superficial generalised	• 24/46 (52.2%)	• 0/1 (0%)	• 24/47 (51.1%)
• Other	• 11/26 (42.3%)	• 2/7 (28.6%)	• 13/33 (39.4%)
• Pyoderma – superficial focal	• 11/25 (44.0%)	• 0	• 11/25 (44.0%)
• Dermatitis – *Malassezia*	• 15/22 (68.2%)	• 0/1 (0%)	• 15/23 (65.2%)
• Open diagnosis	• 6/16 (37.5%)	• 0/2 (0%)	• 6/18 (33.3%)
• Dermatitis – food allergy	• 2/16 (12.5%)	• 0/1 (0%)	• 2/17 (11.7%)
• Pyoderma – deep	• 16/16 (100%)	• 0	• 16/16 (100%)
• Pemphigus foliaceus	• 2/11 (18.2%)	• 1/3 (33.3%)	• 3/14 (21.4%)
• Cutaneous neoplasia	• 1/10 (10.0%)	• 0	• 1/10 (10.0%)
• Pyoderma – surface	• 4/7 (57.1%)	• 0/1 (%)	• 4/8 (50.0%)
• Sebaceous adenitis	• 1/5 (20.0%)	• 0	• 1/5 (20.0%)
• Dermatitis – flea allergy	• 1/4 (25.0%)	• 0	• 1/4 (25.0%)
• Dermatophytosis	• 2/2 (100%)	• 0/2 (0%)	• 2/4 (50.0%)
• Cushing’s disease	• 0/3 (0%)	• 0	• 0/3 (0%)
• Demodicosis	• 0/3 (0%)	• 0	• 0/3 (0%)
• Alopecia X	• 0/2 (0%)	• 0	• 0/2 (0%)
• Cellulitis	• 1/2 (50.0%)	• 0	• 1/2 (50.0%)
• Cutaneous dysbiosis/bacterial or yeast overgrowth	• 2/2 (100%)	• 0	• 2/2 (100%)
• Abscess	• 0/1 (0%)	• 0	• 0/1 (0%)
• Cutaneous lupus erythematosus	• 0	• 0/1 (0%)	• 0/1 (0%)
• Hair follicle dysplasia	• 0/1 (0%)	• 0	• 0/1 (0%)
• Sterile nodular panniculitis	• 1/1 (100%)	• 0	• 1/1 (100%)
• Systemic fungal infection	• 0/1 (0%)	• 0	• 0/1 (0%)
• Wound – bite	• 1/1 (100%)	• 0	• 1/1 (100%)
• Wound – traumatic	• 0/1 (0%)	• 0	• 0/1 (0%)
**Otic (ear)**	136/211 (64.5%)	12/31 (38.7%)	148/242 (61.2%)
• Otitis externa	• 113/175 (64.6%)	• 5/15 (33.3%)	• 118/190 (62.1%)
○ Caused by allergy of unknown aetiology	○ 38/66 (57.6%)	○ 0	○ 38/66 (57.6%)
○ Caused by atopic dermatitis (environmental allergy)	○ 37/53 (69.8%)	○ 0/2 (0%)	○ 37/55 (67.3%)
○ Caused by autoimmune condition (e.g. pemphigus foliaceus)	○ 1/1 (100%)	○ 0	○ 1/1 (100%)

○ Caused by bacteria<br>○ Caused by food allergy<br>○ Caused by yeast<br>○ No bacteria, parasites or yeast detected<br>○ Other<br>○ Open diagnosis	○ 51/65 (78.5%)<br>○ 9/13 (69.2%)<br>○ 46/58 (79.3%)<br>○ 0/3 (0%)<br><br>○ 3/3 (100%)<br>○ 0/1 (0%)	○ 5/10 (50.0%)<br>○ 0<br>○ 0/2 (0%)<br>○ 0<br><br>○ 0/4 (0%)<br>○ 0/1 (0%)	○ 56/75 (74.7%)<br>○ 9/13 (69.2%)<br>○ 46/60 (76.7%)<br>○ 0/3 (0%)<br><br>○ 3/7 (42.9%)<br>○ 0/2 (0%)
• Otitis media<br>• Other<br>• Open diagnosis<br>• Otitis interna<br>• Not recorded in medical record	• 10/14 (71.4%)<br>• 4/8 (50.0%)<br>• 6/10 (60.0%)<br>• 1/1 (100%)<br>• 2/3 (66.7%)	• 3/6 (50.0%)<br>• 1/4 (25.0%)<br>• 1/1 (100%)<br>• 2/5 (40.0%)<br>• 0	• 13/20 (65.0%)<br>• 5/12 (41.7%)<br>• 7/11 (63.6%)<br>• 3/6 (50.0%)<br>• 2/3 (66.7%)
**Interdigital**<br>• Dermatitis – atopic (environmental allergy)<br>• Dermatitis – *Malassezia*<br>• Dermatitis – allergy of unknown aetiology<br>• Pyoderma<br>• Interdigital furunculosis/cysts<br>• Other<br>• Dermatitis – food allergy<br>• Not recorded in medical record<br>• Open diagnosis	32/77 (41.6%)<br>• 8/18 (44.4%)<br>• 6/16 (37.5%)<br>• 2/15 (13.3%)<br>• 7/10 (70.0%)<br>• 6/8 (75.0%)<br>• 2/6 (33.3%)<br>• 0/2 (0%)<br>• 0/1 (0%)<br>• 1/1 (100%)	0/2 (0%)<br>• 0<br>• 0<br>• 0<br>• 0<br>• 0<br>• 0/2 (0%)<br>• 0<br>• 0<br>• 0	32/79 (40.5%)<br>• 8/18 (44.4%)<br>• 6/16 (37.5%)<br>• 2/15 (13.3%)<br>• 7/10 (70.0%)<br>• 6/8 (75.0%)<br>• 2/8 (25.0%)<br>• 0/2 (0%)<br>• 0/1 (0%)<br>• 1/1 (100%)
**Ear pinnae**<br>• Dermatitis – atopic (environmental allergy)<br>• Dermatitis – allergy of unknown aetiology<br>• Open diagnosis<br>• Vasculitis<br>• Other	5/13 (38.5%)<br>• 3/6 (50.0%)<br>• 0/2 (0%)<br>• 1/2 (50.0%)<br>• 0/2 (0%)<br>• 1/1 (100%)	0/1 (0%)<br>• 0<br>• 0/1 (0%)<br>• 0<br>• 0<br>• 0	5/14 (35.7%)<br>• 3/6 (50.0%)<br>• 0/3 (0%)<br>• 1/2 (50.0%)<br>• 0/2 (0%)<br>• 1/1 (100%)
**Perianal/perineal**<br>• Perianal fistula<br>• Anal sacculitis<br>• Dermatitis – allergy of unknown aetiology<br>• Dermatitis – *Malassezia*	4/10 (40.0%)<br>• 1/5 (20.0%)<br>• 2/3 (66.7%)<br>• 0/1 (0%)<br>• 1/1 (100%)	1/1 (100%)<br>• 0<br>• 0<br>• 1/1 (100%)<br>• 0	5/11 (45.5%)<br>• 1/5 (20.0%)<br>• 2/3 (66.7%)<br>• 1/2 (50.0%)<br>• 1/1 (100%)
**Nasal planum**<br>• Other<br>• Discoid lupus erythematosus<br>• Nasal hyperkeratosis<br>• Open diagnosis<br>• Vasculitis	3/9 (33.3%)<br>• 1/3 (33.3%)<br>• 1/3 (33.3%)<br>• 0/1 (0%)<br>• 0/1 (0%)<br>• 1/1 (100%)	1/2 (50.0%)<br>• 1/2 (50.0%)<br>• 0<br>• 0<br>• 0<br>• 0	4/11 (36.4%)<br>• 2/5 (40.0%)<br>• 1/3 (33.3%)<br>• 0/1 (0%)<br>• 0/1 (0%)<br>• 1/1 (100%)
**Nailbed**	2/8 (25.0%)	0/1 (0%)	2/9 (22.2%)

• Bacterial onychitis<br>• Malassezia paronychia<br>• Lupoid onychitis/lupoid onychodystrophy<br>• Pemphigus foliaceus	• 1/3 (33.3%)<br>• 1/3 (33.3%)<br>• 0/2 (%)<br>• 0	• 0<br>• 0<br>• 0<br>• 0/1 (0%)	• 1/3 (33.3%)<br>• 1/3 (33.3%)<br>• 0/2 (0%)<br>• 0/1 (0%)
**Mucocutaneous junctions**<br>• Mucocutaneous pyoderma<br>• Other<br>• Open diagnosis	4/5 (80.0%)<br>• 2/3 (66.7%)<br>• 1/1 (100%)<br>• 1/1 (100%)	0/1 (0%)<br>• 0<br>• 0/1 (0%)<br>• 0	4/6 (66.7%)<br>• 2/3 (66.7%)<br>• 1/2 (50.0%)<br>• 1/1 (100%)
